# Short-term memory as a working memory control process

**DOI:** 10.3389/fpsyg.2013.00013

**Published:** 2013-01-31

**Authors:** Eddy J. Davelaar

**Affiliations:** Department of Psychological Sciences, Birkbeck College, University of LondonLondon, UK

**A commentary on**

**About the distinction between working memory and short-term memory**

by Aben, B., Stapert, S., and Blokland, A. (2012). Front. Psychology 3:301. doi: 10.3389/fpsyg.2012.00301

Aben et al. (2012) take issue with the unthoughtful use of the terms “working memory” (WM) and “short-term memory” (STM) in the cognitive and neuroscientific literature. Whereas I agree that neuroscientists using the term WM to refer to sustained neural activation and cognitive psychologists using the terms interchangeably reflects that the field has lost control over its own dictionary, the recommendations to develop more tasks does not seem to get to the heart of the matter. Here, I argue in favor of a theoretical approach to the constructs of WM and STM, as the terms have become as impure as the tasks that purport to measure the constructs.

## STM vs. WM

The concepts of STM and WM are theoretical and should be treated like that. In the 1960s, STM was equated to primary memory. Atkinson and Shiffrin (1968) theorized that the cognitive system sets up a buffer to maintain information temporally. This has been interpreted as a structural buffer that maintains temporary information, but could also mean a temporary buffer that maintains durable information. The theory states that “information entering STS comes directly from LTS and only indirectly from the sensory register” (Atkinson and Shiffrin, 1968, p. 115). In other words, “the activation of a feature in LTS is equivalent to the placing of this item in STS” (Shiffrin, 1976, p. 194). Furthermore, the “initial activation of the storage causes temporary traces to appear which dissipate unless some action is taken to maintain them. This activity is primary memory” (Norman, 1968, p. 525). Thus, *STM is a process, not a structure*.

Baddeley (1966) observed that immediate recall was particularly sensitive to interference when maintaining acoustic compared to semantic information, which eventually led to the assumption that verbal STM as a structural component operates on phonological information (Baddeley and Hitch, 1974). The WM model *initially* separated the executive control processes from the short-term storage component. Thus, WM = STM + executive control processes. However, in later work, the process of active maintenance of information was considered one of the executive processes performed by the central executive (Baddeley, 1996) and that the active part of phonological LTM is the content of the phonological loop (Baddeley et al., 1998). This implies that WM = executive control processes or *STM = process within WM*.

## Simple span vs. complex span

Aben et al. suggest that in order to understand the STM/WM distinction more work is needed in which tasks vary in the duration that information is kept and the cognitive load. This is a theory-laden suggestion, as it assumes that forgetting in STM is due to time-based decay and that WM is best assessed by varying cognitive load. This is a hotly debated position (see e.g., Barrouillet et al., 2007; Lewandowsky et al., 2009) and it will be a theoretical nightmare to construct a set of tasks on which all researchers could agree. This is even more aggravated by the fact that short-term forgetting is at the heart of another debate on whether there is a need to postulate a limited-capacity short-term buffer (Crowder, 1982; Greene, 1986; Howard and Kahana, 2002; Davelaar et al., 2005; Brown et al., 2007). Provocatively put, a simple span task may never measure *STM storage capacity*, as STM does not exist!

Nevertheless, those who take the view that there is no such thing as STM still talk about WM (e.g., Brown et al., 2007), suggesting that a general consensus is that *WM includes processes that are not related to short-term storage*.

Another problem with suggesting developing more tasks is that such a focus may fall prey to the criticism of circularity. That is, one creates a “simple” task that only requires storage, a “complex” task that requires additional processes, and then uses these tasks to show differential correlation patterns between the tasks and some cognitive ability. By design, the tasks will follow a WM = STM + X format. Showing that the STM component or the X component correlates with fluid intelligence, reasoning, or language ability, does not say anything about the natures of and distinction between STM of WM. The distinction is already built in by the choice of tasks.

Aben et al. acknowledge that the simplicity of tacking on a secondary task to a simple span task has led to a proliferation of WM tasks. Yet, different WM tasks do not load on a single WM construct (Miyake et al., 2000). The explanation is that WM is composed of distinct processes (Baddeley, 1996; Miyake et al., 2000). However, this defeats the purpose of creating a complex span task to measure WM capacity, as it would require the development of several complex span tasks measuring capacities of “shifting,” “inhibition,” “updating,” “dual tasking,” and so on.

A more severe problem with following labeled statistical latent variables is the disregard of procedural similarity. For example, Engle et al.'s (1999) WM and STM latent constructs are perfectly confounded with the dual-task and single-task requirement of the component tasks, respectively. This is also the case when the modality of presentation is taken into account (Kane et al., 2004). In fact Oberauer et al. (2012), presented a computational model that captures the Kane et al. (2004) data without recourse to different processing capacities. In their model, the statistical latent constructs truly represent different behavioral patterns due to task-procedural differences and not due to different latent cognitive constructs. Hence, developing new complex span tasks would not provide evidence for separate WM and STM constructs.

## Conclusion

The review by Aben et al. highlights a rough edge in the STM/WM literature, but focuses too much on the most impure part of the STM/WM distinction, the span tasks. The distinction between STM and WM and, more importantly, their interrelationship becomes clear at the theoretical level, but task impurity will plague any correlational study. Addressing the distinction at the theoretical level allows honing in on the questions that speak directly to the nature and mechanism of STM/WM and provide directions toward more suitable approaches.
